# Interdisciplinary modelling approaches for One Health in agri-food systems: a scoping review and conceptual analysis

**DOI:** 10.1186/s12889-026-29666-2

**Published:** 2026-10-03

**Authors:** Thomas Art Burke, Antje Risius

**Affiliations:** 1 Department for Agricultural Economics and Rural Development, Georg-August-Universität Göttingen, Platz der Göttinger Sieben 5, 37073 Göttingen, Germany; 2 Department of Nutrition, Division of Sustainable Nutrition and Distribution, University of Applied Sciences Fulda, Leipzigerstr. 123, 36037 Fulda, Germany

**Keywords:** One Health, Modelling, Food, Foodborne, Epidemiology, Systems

## Abstract

**Background:**

Increasingly, the One Health approach must be applied to a changing food system to simultaneously support nutritional, infectious disease, and environmental outcomes. Successful interventions by public health authorities will require quantitative modelling for evaluating trade-offs and prioritization. Herein, we reviewed methodologies for One Health modelling relevant to the food system and proposed a framework for supporting international and national One Health initiatives within governmental institutions.

**Methods:**

This study first identified an operational scope of One Health by reviewing the One Health Joint Plan of Action (OH-JPA) for proposed activities related to the food system. Then we cross-referenced their implementation within the Group of 20 countries through a review of their publicly available One Health resources. We next conducted a PRIMSA scoping review on One Health quantitative modelling with relevance to the food system from 2021 to 2026 to determine interdisciplinary collaboration, data types, and modelling methodologies. Lastly, we developed a conceptual framework for modelling within One Health initiatives involving the food system.

**Results:**

We found a narrow scope of institutional initiatives at the intersection of One Health and the food system. The activities proposed in the OH-JPA and executed in G20 governments focused on coordination, interoperability, and data sharing to support programs on antimicrobial resistance, zoonoses, and vector-borne diseases. The scoping review produced 129 relevant One Health research articles. In addition to epidemiologic data, One Health modelling used agricultural, geospatial, and ecological data for supporting food system topics. Quantitative modelling approaches either used heterogenous data, incorporated mixed effects, or integrated multiple models for scenario analysis. We proposed a modular framework for the operationalization of modelling in institutions to support interdisciplinary collaboration between food systems and One Health research.

**Conclusions:**

One Health is a holistic approach, but in practice is narrowly implemented. Quantitative modelling in One Health reflects the complex dynamics in the animal-human-environment interface. Infrastructure for genomic, geospatial, and agricultural data would facilitate interdisciplinary modelling approaches beneficial for One Health in the food system.

**Supplementary Information:**

The online version contains supplementary material available at https://doi.org/10.1186/s12889-026-29666-2.

## Introduction

Quantitative modelling underlies many of the objectives for One Health, despite that the latter’s focus on coordination and capacity building rather than strictly scientific inquiry. The UN Quadripartite^1^ describes One Health as “… an integrated, unifying approach” and that “.mobilizes multiple sectors, disciplines and communities at varying levels of society” [1]. Integrating this mobilization effectively requires the quantification of disease determinants, intervention effectiveness, and intersectoral trade-offs to achieve equitable outcomes among all stakeholders [2, 3]. However, One Health’s wide definition reduces clarity on which quantitative modelling techniques are relevant to the food system and how they are to be deployed in public health institutions [4].

The food system currently faces multiple crises that require integrated assessments on nutritional and environmental outcomes [5]. The modern food system contributes directly to deforestation, climate change, and biodiversity reduction, and it is also attributed to increasing the incidence of metabolic and cardiovascular diseases [6, 7]. Initiatives, such as the EAT-Lancet commission [5] and the Food Systems Economic Commission [8], quantify these outcomes for potential interventions by combining methodologies from agricultural economics, public health nutrition, and planetary science. Proposed changes to food systems may interact with One Health objectives by changing disease exposures or production environments, of which quantitative modelling could clarify potential hazards or co-benefits.

Many drivers of One Health issues are modulated by the structure of food systems, yet the role of quantitative modelling in One Health initiatives is underdeveloped. Studies and commissions have recognized food systems as a crucial mediator at the interface between humans, livestock, wildlife, and the environment, but they have not articulated this in the working processes of One Health [9, 10]. A previous review by Arredondo-Rivera and colleagues found only 2 relevant modelling studies, but this may be due to disciplinary siloing between food systems and One Health [11]. A review that uses a methodologically focused and policy-grounded search criteria may reveal further bridges between One Health initiatives and the food system.

This study determined the current working scope of One Health in food systems within international and national One Health initiatives and reviewed the corresponding mathematical and statistical modelling techniques. Because of international alignment on One Health as an actionable framework, we distinguished between real-world implementations and theoretical applications to derive a framework for One Health in food systems. We anticipate the findings to clarify public health infrastructure priorities for policymakers and to add to discourse on the definition and application of One Health as a concept in public health research.

## Methods

The study had three data sources: an analysis of the Quadripartite OH-JPA, public materials on One Health initiatives of the G20 nations, and a scoping review on One Health quantitative modelling. We conducted a policy analysis of the One Health Joint Plan of Action (OH-JPA) by the UN Quadripartite and cross-referenced the implementation of its food-system activities to national One Health initiatives within the governments of the Group of 20 (G20) nations. This formed an operational definition of One Health in the food system that we applied to a scoping review on quantitative modelling methodologies. From these data, we formed a framework for the role of quantitative modelling within administrative apparatuses to support collaboration, coordination, and interdisciplinary exchange between One Health and the food system.

### OH-JPA analysis

We first assessed the OH-JPA to determine international priorities of One Health activities, especially in the aftermath of the COVID-19 pandemic [1]. The OHJPA is divided into 6 Action Tracks, sub-divided into “actions”, with further specification of activities and deliverables. In our analysis of the OH-JPA, we identified the activities that had relevance to food systems (i.e. directly involving food, livestock, and/or agricultural land usage). We then created a matrix of One Health priorities in the food system classified by exposures, populations, pathogens, and research approaches (Table S1).

### One health institutions in G20 nations

We gathered online resources on One Health initiatives by G20 public health agencies to find example implementations of the OH-JPA embedded in national governments. The G20 represent about 75% of global trade, every populated continent, and 56% of the population of the world [12], providing a robust sample of governments implementing One Health policies.

For each country, we performed a web search for publicly visible One Health initiatives in national governments. We conducted a top-level domain search for each government (e.g., site:.gov) for the key phrase “One Health” and the equivalent translated term, using both Google and DuckDuckGo search engines. This search strategy is intended to find existing current One Health units and initiatives as of June 2026. For each country, we documented One Health landing pages, the agencies/ministries involved, the governance structure of One Health activities, and the responsibilities and priorities (Table S6). We cross-referenced these priorities with the OH-JPA matrix.

### One health modelling scoping review

We conducted a scoping review of One Health modelling methodologies relevant to the food system based upon the priorities identified by our analysis of the OH-JPA and the G20. We conducted the review based on the Preferred Reporting Items for Systematic Reviews and Meta-Analyses for Scoping Reviews (PRISMA-SR) guideline^2^ [13]. For the scoping review, we chose PubMed as the primary database, with Google Scholar and Web of Science as supplemental databases for supporting literature. The search used Boolean join between “One Health” and “Modelling” with an additional AND subject from a nested OR list of each institutional priority. We searched titles and abstracts, using both MeSH terms and free form text. In the search criteria, we excluded papers labelled as reviews in PubMed. In addition, we filtered for English only articles with full-text availability. The dates are limited to January 01, 2021, to May 31, 2026. The full search criteria are below:

- ((“One Health“[MeSH] OR “One Health“[tiab]) AND model*[tiab]) AND
- ((Food Safety[MeSH] OR “Food Safety“[tiab]) OR
- (Emerging Infectious Diseases[MeSH] OR “emerging infectious disease“[tiab]) OR
- (Zoonoses[MeSH] OR zoonotic[tiab]) OR
- (Vector-Borne Diseases[MeSH] OR “vector-borne“[tiab]) OR
- (Neglected Tropical Diseases[MeSH] OR “neglected tropical disease“[tiab]) OR.
- (water[tiab] AND hygiene[tiab]) OR.
- (Disease Surveillance[MeSH] OR surveillance[tiab]) OR.
- (pandemic[tiab] AND (preparedness[tiab] OR response[tiab])) OR.
- (Drug Resistance, Microbial[MeSH] OR “antimicrobial resistance“[tiab])) NOT (review[pt])

We used a multi-criteria screening process to narrow the results to relevant studies of One Health modelling within the food system. The first filter removed articles that were perspectives or reviews. The second filter was topical, sorting studies into 7 categories: laboratory-based; exclusively bioinformatics-based; analyses on One Health coordination, concepts, and stakeholders; cross-sectional and prevalence; modelling studies with food-systems relevance; modelling studies without food-systems relevance; and irrelevant studies (Table S1). Sorting was verified using two open-weight Large Language Models (Meta Llama 3.1 8b Instruct and OpenAI GPT OSS 120b) hosted by the Gesellschaft für Datenverarbeitung mbH Göttingen (GWDG). The service allows the usage of locally hosted LLMs for natural language processing. The sorter gave a master prompt that supplied Table S1 and instructed the LLMs to assign each title/abstract a category. Lastly, we compared the verification sorting with the human sorting. For any discordant pairs, we used a reconciliation process with the other co-author to determine sorting.

We extracted data from the One Health modelling studies through a manual review process with an accompanying verification using LLMs. We extracted modelling domains, a free-text methods description, data types, One Health pillar (e.g. humans, livestock), diseases/infectious agents, and location of the study population (if applicable). For the extraction, we first reviewed abstracts and then followed up with a review of methods sections in articles where there was not sufficient clarity. We used a macro-enabled Excel spreadsheet to classify the modelling domain, data types, and One Health pillar categories. The LLM verification followed a similar procedure from the screening process (using tables S2 – S4) with follow-up reconciliation with the second author.

We synthesized the results of the scoping review with descriptive statistics and a network analysis of the extracted data. We aggregated modelling domains, diseases, and data types using the pandas library in Python. We constructed graph networks for modelling domains, diseases, and data types using NetworkX. For each graph, we used the extracted data category as the nodes. The edges were constructed if a study had multiple types of a given data category. For instance, if the study used both genomic and spatial data, a connection is built between these two nodes. We calculated the centrality and the approximate current flow betweenness centrality of the nodes within each network. We also constructed a graph network of the connections between modelling domains and data types.

### Conceptual framing of one health modelling

We synthesized a framework for quantitative modelling within One Health administrative infrastructure to accommodate food systems analysis. We used the governance and responsibilities of the G20 One Health initiatives alongside the activities of the OH-JPA to determine information technology resources, stakeholders, and One Health products. The results from the scoping review determined the mechanisms and directionality of the framework’s flow diagram.

## Results

### One health joint plan of action analysis

The OH-JPA outlines several mechanisms for international, cross-institutional coordination and mobilization for One Health planning and execution that are relevant to food systems. Every Action Track (AT) had activities related to the food system, but Action Tracks 2 (Zoonoses), 4 (Food Safety), and 6 (Environmental health) had the greatest number. Action Track 2 advises identifying and prioritizing upstream investigations of zoonoses through land-use planning, biodiversity protection in food systems, improving farm biosecurity, and control of zoonoses in livestock and value chains. Action Track 4 focuses on data integrations to improve surveillance and antimicrobial resistance monitoring in the food system. Lastly, Action Track 6 also recommends improved data system interoperability, particularly to support the transition of sustainable agriculture and livestock production. These actions prioritize both food system actions as well as modelling frameworks to support their planning and execution.

### One Health priorities of countries

Every G20 nation uses One Health approaches in some capacity, but formal adoption of One Health capacities is more limited. Each G20 member has policy briefs, plenary meetings, or other outputs regarding One Health methods. As of 2026, the United States, Brazil, Argentina, Australia, India, Italy, South Korea, and the European Union featured specific offices tasked directly with executing One Health programs (Table 1; Table S6). Additionally, Mexico, Japan, Germany, France, and Canada have inter-ministerial coordinating mechanisms, though no established coordinating office. We could not find substantial online resources for Indonesia, Russia, Saudi Arabia, South Africa, or Turkey.

**Table 1:** Overview of governance, responsibilities, and diseases from the Group of 20 nations with visible One Health initiatives

G20 Country	Governance Description	Responsibilities	Diseases
Argentina	Unit called National Institute of Tropical Medicine within the National Administration of Laboratories and the Institutes of Health. Founded in 2011.	Research, Capacity Building, Advisory, Risk communication, Surveillance	Zoonoses, Neglected Tropical Diseases, Vector-borne, AMR
Australia	Inter-ministerial coordination from Centre for Disease Control with ministries of Agriculture, Environment, and State.	Coordination, Capacity Building, Risk Communication	Zoonoses, Food Safety, Environmental
Brazil	Technical Group of One Health within the Ministry of Health. Established by legislation in 2024.	Capacity building, coordination, Advisory, Research	Zoonoses, Pandemic prevention, food safety, AMR, Environmental
Canada	Inter-ministerial coordination as part of the Federal Health Portfolio	Surveillance, Coordination, Research, Capacity building, Risk communication	AMR, Zoonoses, Food Safety, Environmental
France	Inter-ministerial coordination within the Ministry of Health	Coordination, Research, Surveillance	Antimicrobial Resistance
Germany	Distributed responsibilities among several agencies and government institutes (e.g. Robert Koch Institute, Friedrich Loeffler Institute)	Research, Coordination	Antimicrobial Resistance
India	Unit called National One Health Mission within Department of Health Research, established in 2024.	Capacity building, Coordination, Capacity Building, Surveillance	Pandemic prevention, antimicrobial resistance, zoonoses
Italy	Department of Human Health, Animal Health, and Ecosystem (One Health) and International Relations within the Ministry of Health, established in 2023. One Health Group in the Institute for Superior Health.	Research, Capacity Building, Surveillance, Coordination	Food Safety, Zoonoses, Environmental
Japan	Inter-ministerial coordination between Ministries of Health, Agriculture, and Environment. Activities began in 2015.	Surveillance, Interventions, Risk communication, Coordination	Zoonoses, Food Safety, Vector-bone
Mexico	Inter-ministerial coordination between health agencies of human health, food safety, animal health, and environmental health.	Coordination	Zoonoses
South Korea	Unit called One Health AMR within National Institute of Health	Research, coordination, capacity building	Antimicrobial Resistance
United States	One Health Office within the Centers for Disease Control and Prevention and coordinates with Departments of Agriculture, Environment, and Interior. Founded in 2009 by legislation.	Coordination, Capacity Building, Advisory, Risk communication, surveillance	Zoonoses, pandemic preparedness, antimicrobial resistance
European Union	Inter-ministerial task force	Research, coordination, capacity building	None specified

The priorities within One Health units focused on infectious diseases in alignment with the OH-JPA. After analysing publicly accessible online materials on One Health from each G20 nation, we found that One Health applications focused on nine topic areas: Food Safety, Zoonotic Disease, Emerging Infectious Disease (EID), Neglected Tropical Disease (NTDs), Vector-borne Disease, Water Hygiene, Infectious Disease Surveillance, Pandemic Preparedness and Response, and Antimicrobial Resistance. These align with the infectious disease mandates in the OH-JPA, but many countries focus only on one or two topics, primarily antimicrobial resistance and zoonoses. Modelling and data infrastructure supports, despite their inclusion in the OH-JPA, do not appear to be central to administrative responsibilities in the G20 One Health initiatives other than subject matter expert availability and coordination mechanisms for research.

### One health methodologies and modelling techniques: scoping review results

**Fig. 1:**
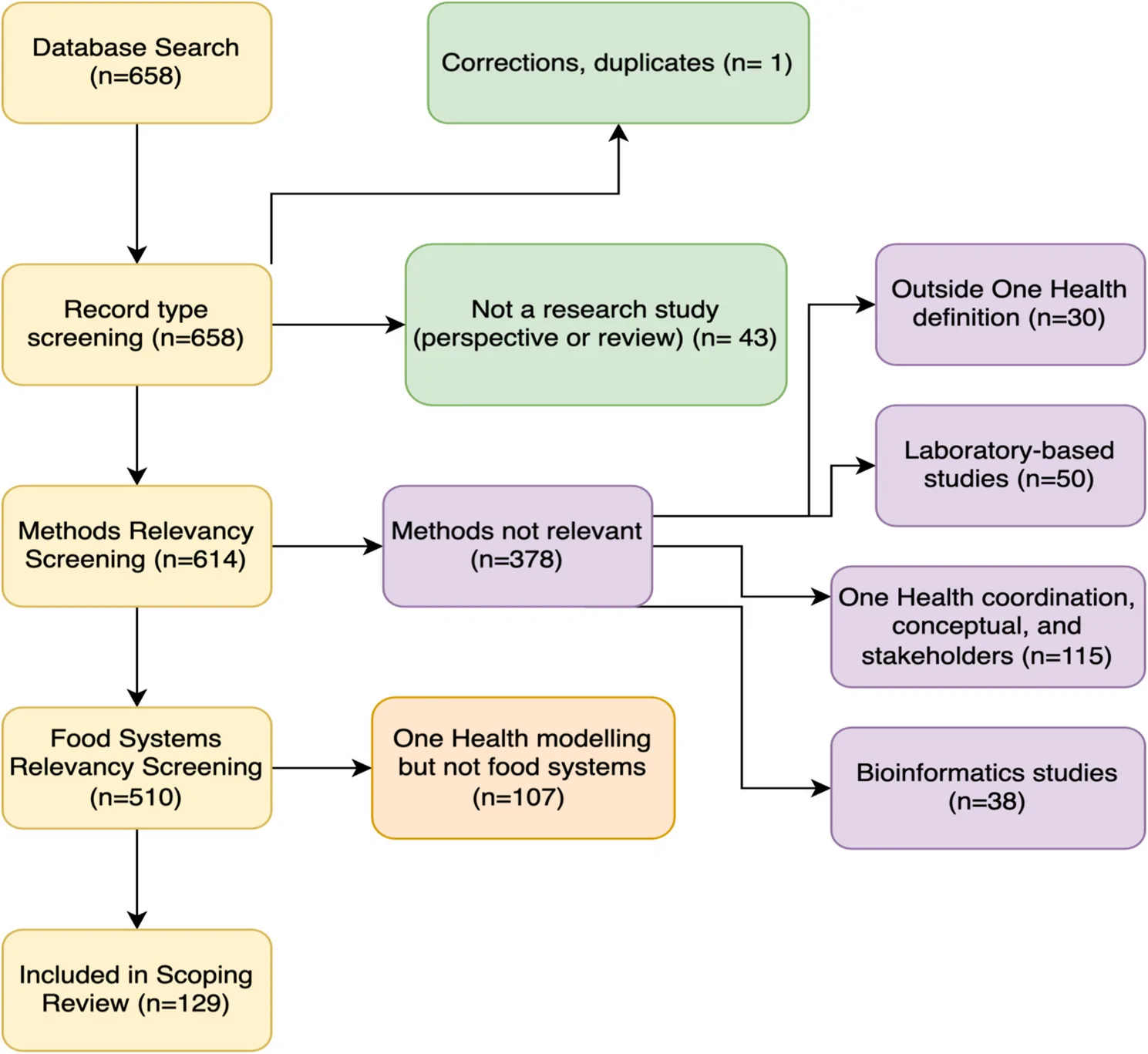
Multi-criteria scoping review screening protocol based on PRIMSA-SR. The yellow steps indicate the records remaining after each classification. The green steps are technical or study type criteria. The purple are studies with non-modelling methodologies. The orange are modelling studies that do not directly involve the food system

The database search began with a total of 658 records and, after multi-criteria sorting, yielded 129 records of One Health modelling studies with relevance to the food system (Fig. 1). In the article type and technical screening, we excluded 31 reviews, 12 perspectives, and 1 duplicate. We then sorted the remaining 615 records into 7 categories based on research study type.

We found 50 laboratory-based studies, 38 bioinformatics studies, 115 One Health coordination and conceptual studies, and 30 studies without relevance to our One Health scope. After this sorting, 236 records were found to be on One Health modelling. We sub-divided these into studies with relevance to the food system (129) and those without relevance (107).

The data extraction found most One Health modelling studies were highly interdisciplinary and integrated several data types together. The dominant modelling domains were epidemiologic (82) and environmental (50), and spatial (38) (Table S7). The top data types were environmental (62), agricultural (54), and disease counts (50) (Table S8). In One Health pillars, Livestock and Humans were the two highest with 87 and 79 studies, respectively (Table S9). Among infectious disease topics, antimicrobial resistance was the most common with 45 studies, followed by Influenza A and E. coli with 11 each (Table S10).

**Fig. 2:**
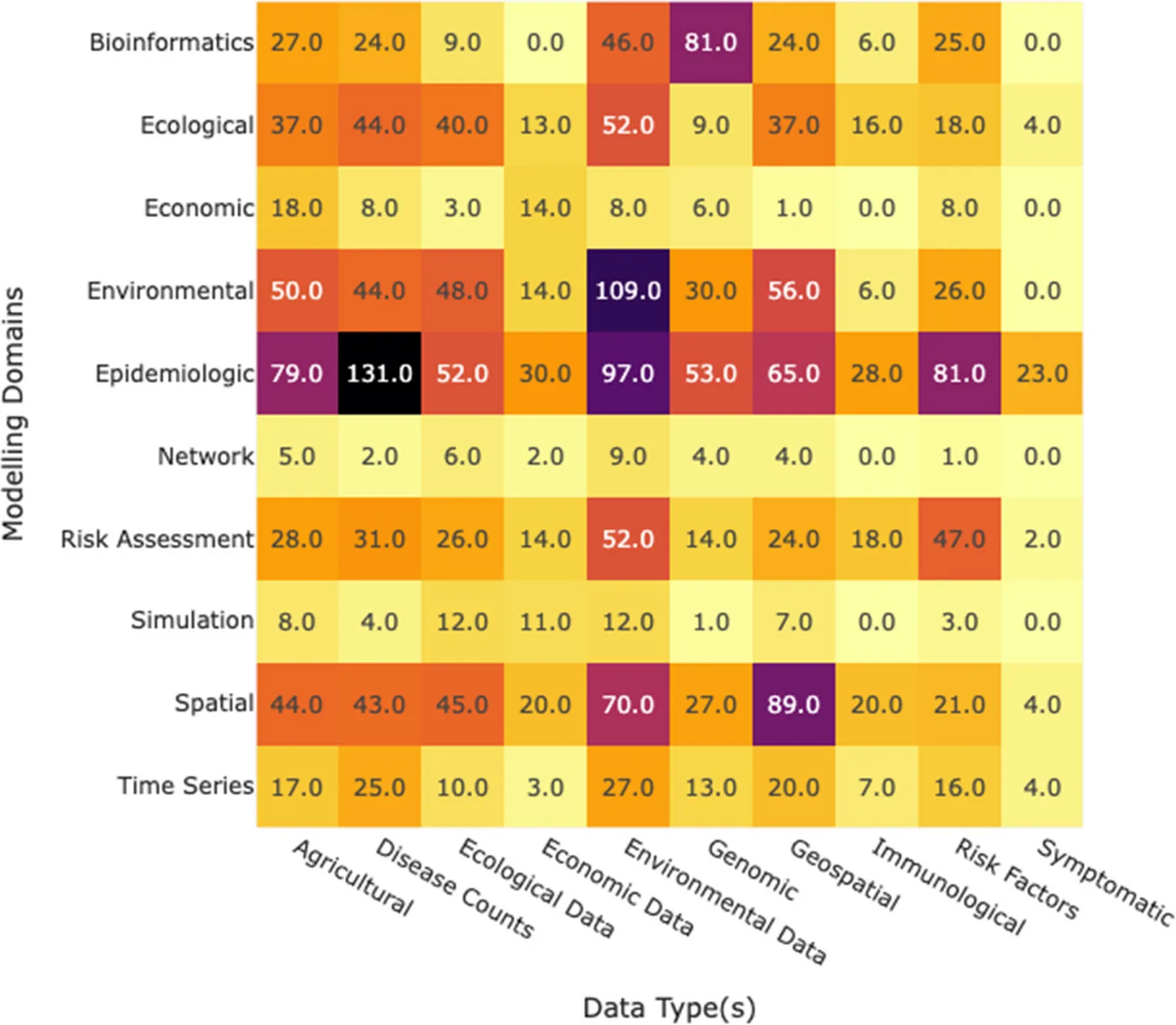
Pair-wise combinations of modelling domains and data types in scoping review studies, depicted as a heat map

**Fig. 3:**
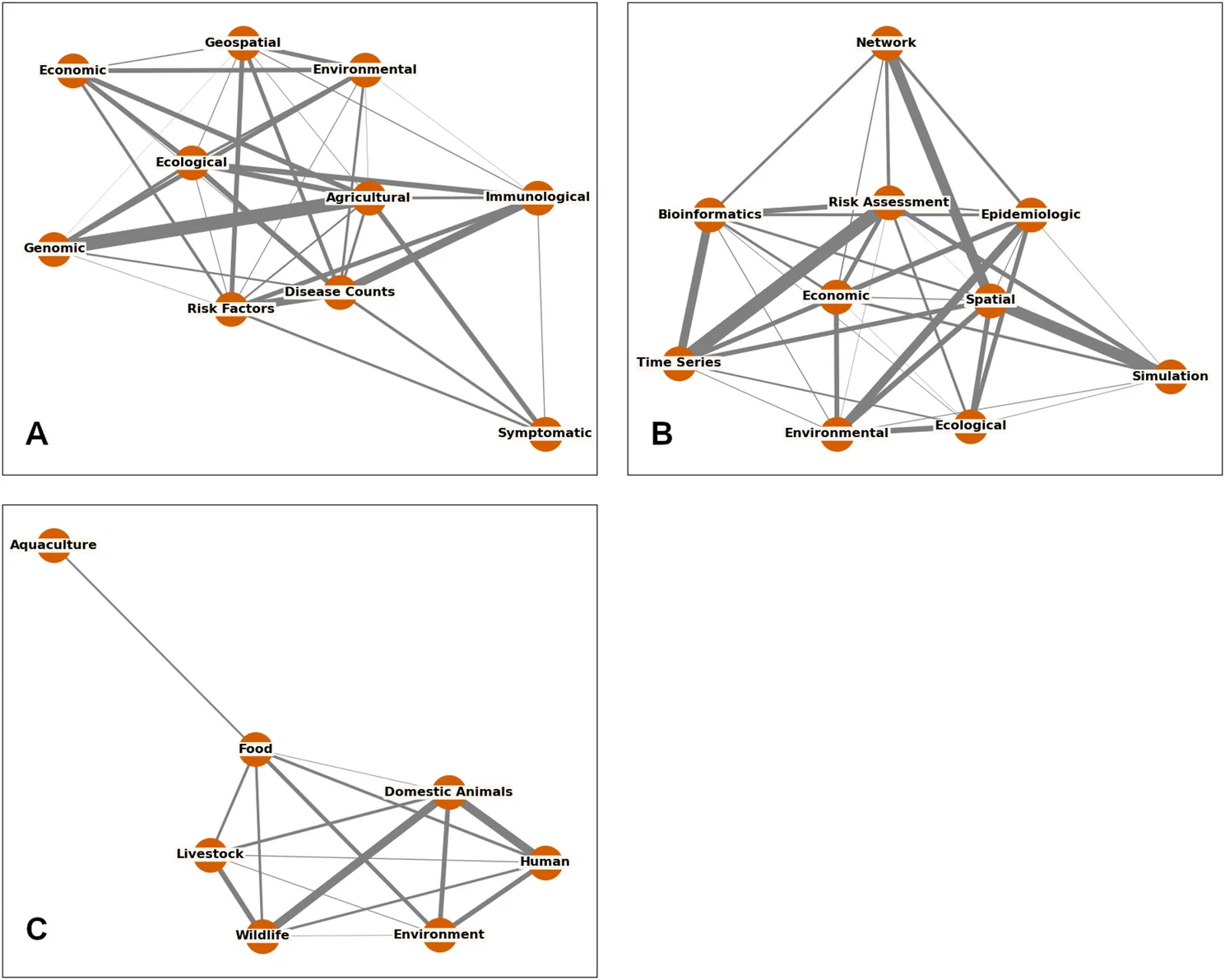
Graph networks of the data extracted from the scoping review articles, with (**A**) depicting the data types, (**B**) depicting the modelling domains, and (**C**) depicting the One Health pillars. Width of edges correspond to number of connections

**Fig. 4:**
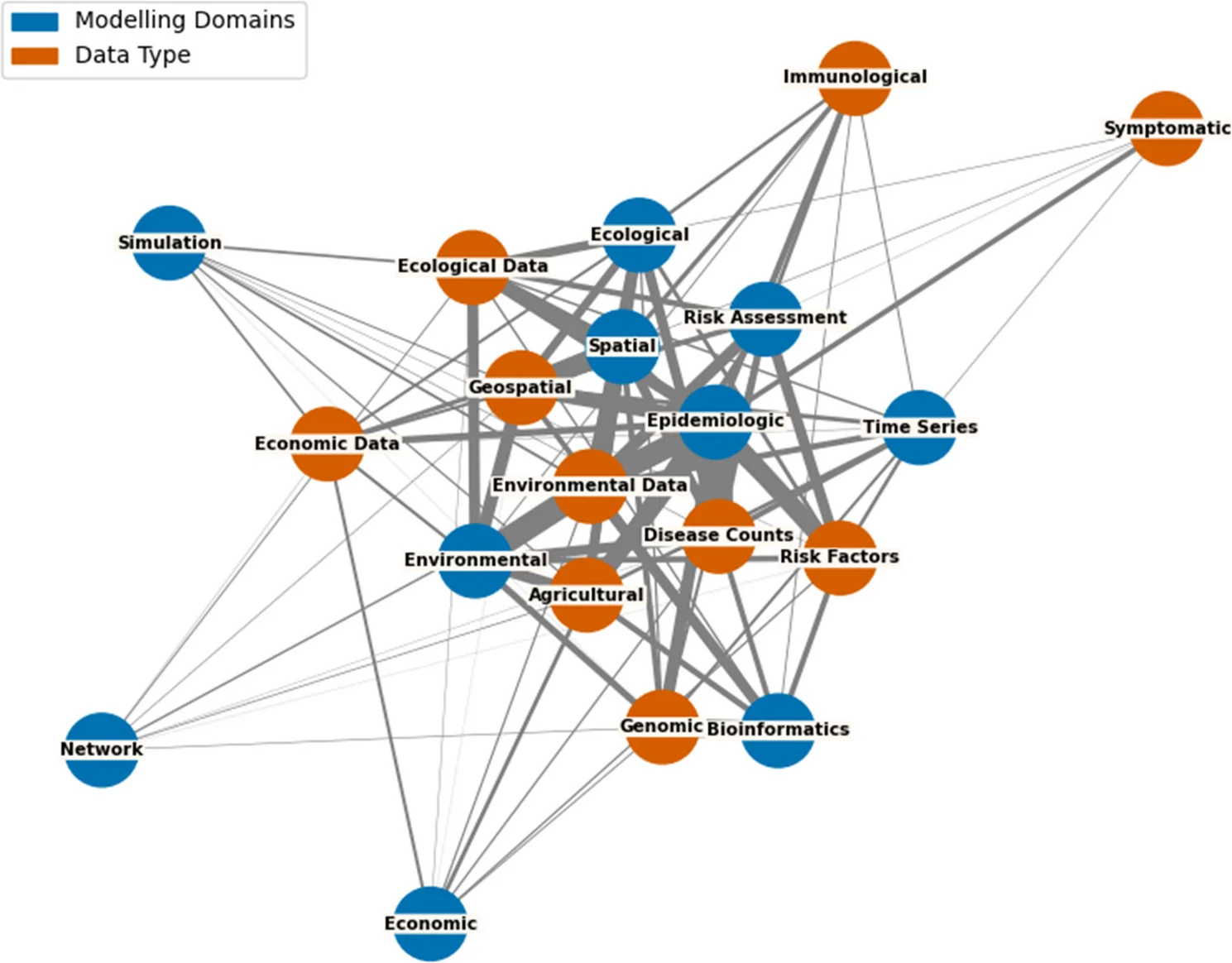
Graph network depicting connections between modeling domains (blue) and data types (orange). Width of edges correspond to number of connections

We examined the interdisciplinarity of the modelling methodologies through pair-wise comparisons and network analysis. Using pair-wise counts (Fig. 2), epidemiology and environmental modelling frameworks had the widest range of data types used. Environmental, geospatial, and ecological data had the widest ranges of modelling domains used. In graph networks (Figs. 3 and 4), we evaluated the centrality (C) of nodes (degree of connectivity to other nodes) and the approximate current flow betweenness centralities (CF) (similar to centrality but incorporates edge weights)^3^. Both centrality measures found food was the most central One Health pillar (C 1.0, CF 0.41), followed by livestock (0.83, 0.19) and the environment (0.83, 0.15). All other One Health pillars also had high centrality except for aquaculture. Agricultural (1.0, 0.17), disease counts (1.0, 0.17), and risk factors (1.0, 0.16) were the most central data types, with genomic and economic data having lower centrality. In modelling domains, economic (1.0, 0.16), epidemiologic (1.0, 0.15), and risk assessments (1.0, 0.14) were the most central. Network and simulation modelling domains had relatively low centrality.

### One health modelling techniques and data modalities

Quantitative modelling for One Health studies used either heterogenous data, mixed effects, or integrative modelling to accommodate the complex dynamics within the food system. The most frequently used modelling framework was generalized linear models, which used many approaches and data types. For instance, El Ghassem and colleagues used a mixed effects logistic regression to integrate data on human and livestock seroprevalence, tick distribution and species, genomic data, and geospatial factors and agricultural production data to assess One Health risks for Crimean-Congo Haemorrhagic Fever [14]. General Additive Models and joinpoint regressions were used to handle mixed effects within a single model, such as Palmeirim and colleagues, who evaluated how indigenous lands and landscape structures shape zoonotic risks [15]. Bayesian approaches, especially hierarchical models, were frequently used to model heterogenous data and gaps in information [16–18]. Machine learning techniques from unsupervised techniques to neural networks were especially useful for integrating genomic and spatial data into epidemiology and environmental studies [19–21]. Integrated modeling tended to use equilibrium-based simulations to project scenarios and combined multiple data types. Process-based mathematical approaches, on the other hand, used compartment models (i.e., systems of differential equations used to simulate pathogen transmission), such as Castonguay and colleagues who projected the impacts of climate and land use change on future avian influenza suitability [22]. Other simulations used agent-based models to simulate interactions between livestock and humans [23, 24]. In ecologically focused studies, researchers developed data-driven favorability models that predicted preferable living conditions for vectors [25, 26]. Despite the diversity of approaches, each study had commonality in handling heterogenous One Health relationships through combining different data types, distributions, and/or models together.

### Synthesis and administrative framing

**Fig. 5:**
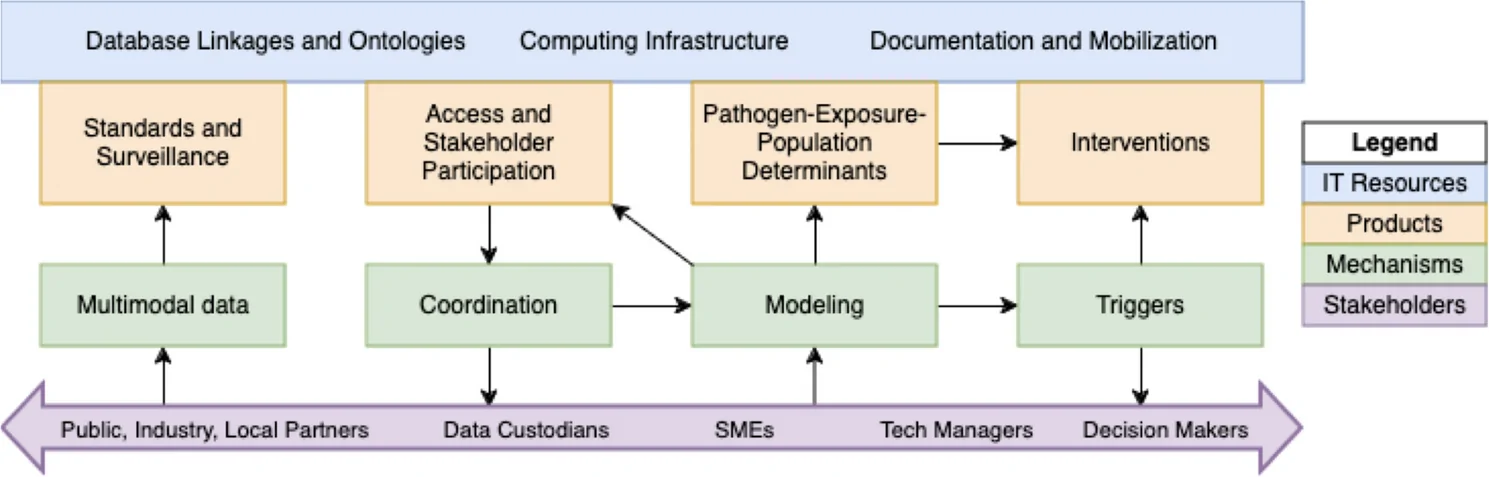
Conceptual framework of embedding One Health modelling into governance structures. Information Technology resource recommendations connect directly to products outlined by the OH-JPA. The connections between mechanisms, products and stakeholders were synthesized from the findings of the scoping review data extraction

We combined the data sources to synthesize the One Health priorities by the OH-JPA and G20 institutions and their constituent quantitative modelling components. We found a small set of actions and infectious diseases that formed most of the One Health overlap with the food system (Tables 2 and 3). The modelling domains and approaches found in the scoping review were used across these actions and infectious diseases. Therefore, administrative structures must accommodate multiple modelling paradigms that enable ad hoc data combinations and interdisciplinary collaborations. Within governance and administrative structures, quantitative models form a loop of stakeholder collaboration among heavy data users that reinforces cooperation and bottom-up surveillance. At the higher levels of hierarchy, conclusions drawn from quantitative models allows for interventional assessment, thereby giving policymakers the ability to anticipate systemic trade-offs and trigger points for public health action (Fig. 5).

**Table 2:** Synthesized recommendations of One Health modelling approaches within governance structures

G20 National One Health Priorities	Administrative Task Description	Administrative Support Methods	Mathematical Models	Minimum One Health Governance
Surveillance Systems	Surveillance are public health information systems tasked with signal detection of emergent pathogens.	IT infrastructure, coordination, capacity building	Autoregressive Integrated Moving Average, Bayesian methods, Machine learning, Integrated modelling	Office
Pandemic Preparedness and Response	Multiple pathogens possible, but emphasis on widespread immune vulnerability and significant morbidity and mortality.	Coordination, research, capacity building	Favourability modelling, Agent-based, Compartmental models, Integrated modelling	Inter-ministerial

**Table 3:** One Health topics among G20 institutions within food systems and their modelling approaches

G20 National One Health Priorities	Route of Transmission	One Health Pillars	Example Pathogens	Mathematical Modelling
Food Safety	Faecal-oral infection route.	Food, Livestock, Wildlife, Humans, Environment	Salmonella spp., E. coli, Campylobacter, Taenia solium, Vibrio spp., Fasciola hepatica	Risk assessments, generalized linear models, mixed effects models, Bayesian methods, machine learning
Zoonotic Disease, Vector-borne, Neglected Tropical Disease	Faecal-oral, respiratory, fomites, or vector-based.	Humans, Animals, Wildlife, Domestic Animals, Livestock, Environment	Influenza, Leptospira spp., Brucella spp., Rabies, Bacillus anthracis, Coxiella burnetii, Crimean-Congo haemorrhagic fever, Mycobacterium avium, Onchocerciasis, Schistosomiasis, Trypanosoma spp., Plasmodium falciparum, West Nile Virus, Hantavirus, Babesia spp.	Favourability models, generalized linear models, mixed effects models, Bayesian methods, compartment model
Antimicrobial Resistance	Primarily bloodstream infections and respiratory diseases. Faecal-oral route also possible.	Humans, Livestock, Environment	E. coli, Acinetobacter, Pseudomonas spp., K. Pneumoniae, Staphylococcus aureus.	Phylogenetics, network analysis, integrated modelling, machine learning, Bayesian methods, generalized linear models, mixed effect models, agent-based

## Discussion

Our findings suggest that modelling One Health within the food system has a modular and hierarchical structure and offers coordinative support to public health actions. Overall, One Health governance concentrates on a narrow set of infectious disease issues, with substantial overlap with the food system. The UN Quadripartite definition of One Health^4^ does not focus only on infectious diseases [1], but in practice, One Health in the food system is confined to food safety, zoonoses, and antimicrobial resistance. Implementations of the OH-JPA within G20 governments used a holistic, multilateral approach, either through a One Health unit/office or through interministerial coordinative processes. The modelling approaches relevant to these issues take modularized approaches, either through incorporating many data types, handling mixed effects within single models, or by creating integrated model structures. One Health institutional structures could improve coordinative and collaborative responsibilities by maintaining data infrastructure to broader groups of academic and governmental researchers.

The OH-JPA integrates many food systems interventions into its recommendations for improving One Health. Their recommendations often coincide with negative externalities from animal-related agriculture, such as biodiversity, land use, and sustainability, that are extensively modelled in food systems research [7, 27, 28]. Contrary to food systems discourse on co-benefits for health and sustainability [5], One Health in the OH-JPA narrowly focuses on infectious disease prevention, detection, and mitigation. In its recommendations for economic analyses, the OH-JPA primarily focuses on cost-benefits of interventions through the alleviation of infectious disease burdens rather than broad economic transformations through food system changes. This topical focus may prevent additional tools and interventions from being considered, particularly demand-side changes like the reduction of animal-source food consumption – incidentally creating siloes in One Health assessments.

Four years after the release of the OH-JPA, there is clear implementation by the G20 nations, though often narrow in disease coverage or operational capacities. The United States, India, and Italy have extensive One Health units with responsibilities that span many of the activities recommended the OH-JPA for the food system. It may not be a coincidence that these most extensive offices have legislative authorization for their establishment or continuing missions [29–31]. It is possible that governmental systems (i.e., parliamentary versus presidential) influence whether countries opt for inter-ministerial One Health mechanisms versus permanent offices. Political mandates may influence the limited topical foci in the G20; multiple nations prioritized antimicrobial resistance as the primary or exclusive issue for One Health. These national priorities were reflected in the scoping review as well, where we found antimicrobial resistance as the leading topic of One Health models.

Our scoping review reflected a small but maturing modelling ecosystem for One Health in the food system with several successful approaches to evaluating infectious disease dynamics between livestock, wildlife, humans, the environment, and food. More established statistical modelling approaches commonly employed by epidemiologists [32], such as generalized linear models, have the capability of using multiple data types and sources to evaluate relevant and novel One Health research questions. Disciplines outside of epidemiology, particularly ecology, economics, and bioinformatics, incorporated their methodological approaches and data types to improve the prediction of reservoir locations in ecological niches [22], structural drivers of antimicrobial use [33], or modelling of pathogen transmission through phylogenetics [34]. Our analysis showed a potential underusage of food as a One Health component and economics as a modelling domain, due to their relatively few studies but high degree of centrality. While the methodological tools exist, modelling could have a greater role in decision making, if they are positioned better in One Health operational processes.

The role of One Health modelling in the food system in public health governance connects localized stakeholders, siloed data repositories, and interventional actions. The consequences of the COVID-19 pandemic emphasized the importance of a systems-based approach to reduce administrative siloing, including for the food and agricultural system [1, 3, 35, 36]. Despite often being executed by research centres or universities, the products of One Health modelling feed into stakeholder relations and the determination of public health actions by regulators. Therefore, modularity within One Health institutions for data support, coordinative structures, and research collaboration is essential to reflect the modularity found in modelling approaches. Enabling ad hoc analysis capabilities can coincide with advances in the uses of artificial intelligence in public policy [37]. The OH-JPA has been successful in improving national initiatives within the G20, but further activities supporting interoperability and research collaboration would more fully enact activities recommended by the UN Quadripartite. Developing empirical infrastructure for the mobilization of governmental and institutional responses will be best served by balancing relative risks between systemic factors while enabling food systems researchers to advise on outcomes in nutrition and environmental impacts.

When considering how to define the concept of “One Health”, our institutional assessment showed that it is a paradigm almost exclusively for infectious disease management. The idea of integrated disease management between animals, humans, and the environment is not new [38], but its recent usage has spurred policy developments and changes to public health practice. The focus on interconnectedness reorients how we use science to address our shared health with nature [39]. Taken together, One Health is defined by its activities, scientifically grounded by the disease ecology between humans, animals, and the environment [1, 40, 41]. The necessity to understand these dynamics is what spurs the One Health approach in practice. We caution to broaden the scope beyond its utility in infectious disease without establishing similar ecological patterns between the One Health pillars.

Our study has limitations in the scope of its data sources. First, we used the OH-JPA as the basis for international policy on One Health programmes, but there are other international bodies that may have relevance for One Health implementation, such as the World Bank. Similarly, we based our study on published literature, but One Health modelling outputs may also be available in government reports and grey literature not indexed in scientific databases. Additionally, we restricted our One Health governmental initiatives to the G20 which is biased towards large, heavily populated, and wealthy countries. We sought to include non-G20 countries in underrepresented regions, such as Nigeria, Ethiopia, Fiji, and Papua New Guinea, but we found few additional One Health initiatives. We chose to more rigidly scope our data sources, because additional ad hoc searching to improve geographic representation and include grey literature would likely add arbitrary bias while negatively affecting reproducibility.

The proposed framework for mathematical modelling takes several assumptions and therefore has limitations. Based on the scope of our data sources, the framework will be best positioned for One Health initiatives in larger and more developed countries. Potential misclassifications during the screening procedure may have influenced the relative importance of genomic and geospatial data sources. Lastly, our derivation of a One Health scope based on policy activities excludes broader interpretations of One Health, which may exclude some modelling techniques in chronic diseases. In sum, the framework is grounded in the consensus activities proscribed by the UN Quadripartite and the contemporary literature on One Health, but its execution assumes adequate infrastructural and methodological capacities exist within national One Health initiatives. The framework is confined to traditional One Health domains of infectious diseases, and hence it anticipates data and modelling techniques relevant for these subjects.

## Conclusion

Reckoning is an apt word to describe public health discourse since the COVID-19 pandemic. As public authorities reform systems to combat future pandemics, the global public health community has rallied around One Health as a means for resolving the complex set of intertwined issues facing humanity in the 21st century [1, 42] The methods and approaches that have fuelled the remarkable public health 20th -century miracle will not necessarily work for the identification, quantification, and intervention for 21st -century public health challenges [43]. Therefore, One Health, as a collaborative methodological framework, is an ideal mechanism for meeting these infectious disease challenges, many of which involve the food system. Our findings found that disciplinary siloing still can occur with this framework, neglecting potential socio-economic, environmental, or cultural trade-offs. Improving the modularity of One Health modelling while also incorporating co-benefits as shared priorities in non-infectious disease domains is one way to improve One Health and food systems simultaneously.

## Supplementary Information

Supplementary Material 1.

Supplementary Material 2.

Supplementary Material 3.

Supplementary Material 4.

## Data Availability

Data generated will be uploaded on the OSF registration page: https://osf.io/qhuyg.

## Footnotes

1 The UN Quadripartite is a One Health consortium between the World Health Organization, the World Organization on Animal Health, the United Nations Environmental Programme, and the Food and Agriculture Organization.

2 Pre-registration under https://osf.io/9ynpr.

3 For full results, see Graph Network Statistics in Supplemental Results.

4 The full definition is “One Health is an integrated, unifying approach that aims to sustainably balance and optimize the health of humans, animals, plants and ecosystems. It recognizes the health of humans, domestic and wild animals, plants and the wider environment (including ecosystems) are closely linked and interdependent. The approach mobilizes multiple sectors, disciplines and communities at varying levels of society to work together to foster well-being and tackle threats to health and ecosystems, while addressing the collective need for clean water, energy and air, safe and nutritious food, taking action on climate change, and contributing to sustainable development.”

## Abbreviations

EID: Emerging Infectious Disease
FAO: Food and Agriculture Organization
G20: Group of 20 Nations
NTD: Neglected Tropical Disease
OH: One Health
OH-JPA: One Health Joint Plan of Action
WHO: World Health Organization
WOAH: World Organization on Animal Health
UNEP: United Nations Environmental Programme
