# Supplementary material for: Interdisciplinary modelling approaches for One Health in agri-food systems: a scoping review and conceptual analysis
Source: BMC Public Health. 2026 Oct 3;26:2760. doi: 10.1186/s12889-026-29666-2 (PMC13633621; doi:10.1186/s12889-026-29666-2)
Supplement: Supplementary file 2 [file 12889_2026_29666_MOESM3_ESM.docx]

**Display Items**

**Table 1.** Overview of Governance, Responsibilities, and Diseases from the Group of 20 Nations with Visible One Health Initiatives

| **G20 Country** | **Governance Description** | **Responsibilities** | **Diseases** |
| --- | --- | --- | --- |
| **Argentina** | Unit called National Institute of Tropical Medicine within the National Administration of Laboratories and the Institutes of Health. Founded in 2011. | Research, Capacity Building, Advisory, Risk communication, Surveillance | Zoonoses, Neglected Tropical Diseases, Vector-borne, AMR |
| **Australia** | Inter-ministerial coordination from Centre for Disease Control with ministries of Agriculture, Environment, and State. | Coordination, Capacity Building, Risk Communication | Zoonoses, Food Safety, Environmental |
| **Brazil** | Technical Group of One Health within the Ministry of Health. Established by legislation in 2024. | Capacity building, coordination, Advisory, Research | Zoonoses, Pandemic prevention, food safety, AMR, Environmental |
| **Canada** | Inter-ministerial coordination as part of the Federal Health Portfolio | Surveillance, Coordination, Research, Capacity building, Risk communication | AMR, Zoonoses, Food Safety, Environmental |
| **France** | Inter-ministerial coordination within the Ministry of Health | Coordination, Research, Surveillance | Antimicrobial Resistance |
| **Germany** | Distributed responsibilities among several agencies and government institutes (e.g. Robert Koch Institute, Friedrich Loeffler Institute) | Research, Coordination | Antimicrobial Resistance |
| **India** | Unit called National One Health Mission within Department of Health Research, established in 2024. | Capacity building, Coordination, Capacity Building, Surveillance | Pandemic prevention, antimicrobial resistance, zoonoses |
| **Italy** | Department of Human Health, Animal Health, and Ecosystem (One Health) and International Relations within the Ministry of Health, established in 2023. One Health Group in the Institute for Superior Health. | Research, Capacity Building, Surveillance, Coordination | Food Safety, Zoonoses, Environmental |
| **Japan** | Inter-ministerial coordination between Ministries of Health, Agriculture, and Environment. Activities began in 2015. | Surveillance, Interventions, Risk communication, Coordination | Zoonoses, Food Safety, Vector-bone |
| **Mexico** | Inter-ministerial coordination between health agencies of human health, food safety, animal health, and environmental health. | Coordination | Zoonoses |
| **South Korea** | Unit called One Health AMR within National Institute of Health | Research, coordination, capacity building | Antimicrobial Resistance |
| **United States** | One Health Office within the Centers for Disease Control and Prevention and coordinates with Departments of Agriculture, Environment, and Interior. Founded in 2009 by legislation. | Coordination, Capacity Building, Advisory, Risk communication, surveillance | Zoonoses, pandemic preparedness, antimicrobial resistance |
| **European Union** | Inter-ministerial task force | Research, coordination, capacity building | None specified |

**Figure 1.** Multi-criteria scoping review screening protocol based on PRIMSA-SR. The yellow steps indicate the records remaining after each classification. The green steps are technical or study type criteria. The purple are studies with non-modelling methodologies. The orange are modelling studies that do not directly involve the food system.

**Figure 2.** Pair-wise combinations of modelling domains and data types in scoping review studies, depicted as a heat map.

**Figure 3.** Graph networks of the data extracted from the scoping review articles, with (**A**) depicting the data types, (**B**) depicting the modelling domains, and (**C**) depicting the One Health pillars. Width of edges correspond to number of connections.

**A**

**B**

**C**

**Figure 4.** Graph network depicting connections between modeling domains (blue) and data types (orange). Width of edges correspond to number of connections.

**Table 2:** Synthesized recommendations of One Health modelling approaches within governance structures.

| **G20 National One Health Priorities** | **Administrative Task Description** | **Administrative Support Methods** | **Mathematical Models** | **Minimum One Health Governance** |
| --- | --- | --- | --- | --- |
| **Surveillance Systems** | Surveillance are public health information systems tasked with signal detection of emergent pathogens. | IT infrastructure, coordination, capacity building | Autoregressive Integrated Moving Average, Bayesian methods, Machine learning, Integrated modelling | Office |
| **Pandemic Preparedness and Response** | Multiple pathogens possible, but emphasis on widespread immune vulnerability and significant morbidity and mortality. | Coordination, research, capacity building | Favourability modelling, Agent-based, Compartmental models, Integrated modelling | Inter-ministerial |

**Table 3.** One Health topics among G20 institutions within food systems and their modelling approaches

| **G20 National One Health Priorities** | **Route of Transmission** | **One Health Pillars** | **Example Pathogens** | **Mathematical Modelling** |
| --- | --- | --- | --- | --- |
| **Food Safety** | Faecal-oral infection route. | Food, Livestock, Wildlife, Humans, Environment | Salmonella spp., E. coli, Campylobacter, Taenia solium, Vibrio spp., Fasciola hepatica | Risk assessments, generalized linear models, mixed effects models, Bayesian methods, machine learning |
| **Zoonotic Disease, Vector-borne, Neglected Tropical Disease** | Faecal-oral, respiratory, fomites, or vector-based. | Humans, Animals, Wildlife, Domestic Animals, Livestock, Environment | Influenza, Leptospira spp., Brucella spp., Rabies, Bacillus anthracis, Coxiella burnetii, Crimean-Congo haemorrhagic fever, Mycobacterium avium, Onchocerciasis, Schistosomiasis, Trypanosoma spp., Plasmodium falciparum, West Nile Virus, Hantavirus, Babesia spp. | Favourability models, generalized linear models, mixed effects models, Bayesian methods, compartment model |
| **Antimicrobial Resistance** | Primarily bloodstream infections and respiratory diseases. Faecal-oral route also possible. | Humans, Livestock, Environment | E. coli, Acinetobacter, Pseudomonas spp., K. Pneumoniae, Staphylococcus aureus. | Phylogenetics, network analysis, integrated modelling, machine learning, Bayesian methods, generalized linear models, mixed effect models, agent-based |

**Figure 5.** Conceptual framework of embedding One Health modelling into governance structures. Information Technology resource recommendations connect directly to products outlined by the OH-JPA. The connections between mechanisms, products and stakeholders were synthesized from the findings of the scoping review data extraction.
