# Supplementary material for: Interdisciplinary modelling approaches for One Health in agri-food systems: a scoping review and conceptual analysis
Source: BMC Public Health. 2026 Oct 3;26:2760. doi: 10.1186/s12889-026-29666-2 (PMC13633621; doi:10.1186/s12889-026-29666-2)
Supplement: Supplementary file 3 [file 12889_2026_29666_MOESM4_ESM.docx]

**Interdisciplinary Modeling Approaches for One Health in Agri-Food Systems: A Scoping Review and Conceptual Analysis**

**Supplementary Material**

Table of Contents

[Supplemental Methods	2](#_Toc239166495)

[Table S1: Scoping Review Topical Screening Criteria	2](#_Toc239166496)

[Table S2: Data Extraction Criteria for Modelling Domains	3](#_Toc239166497)

[Table S3: Data Extraction Criteria for Data Types	3](#_Toc239166498)

[Table S4: Data Extraction Criteria for One Health Pillars	4](#_Toc239166499)

[Supplemental Results	5](#_Toc239166500)

[OH-JPA Activities Relevant to the Food System	5](#_Toc239166501)

[Table S5: Food System - One Health Priorities with OH-JPA Cross-Reference	8](#_Toc239166502)

[Table S6: Group of 20 National One Health Initiatives	10](#_Toc239166503)

[Table S7: Counts of Data Types Used in Scoping Review Studies	18](#_Toc239166504)

[Table S8: Count of Modelling Domains Used in Scoping Review Studies	18](#_Toc239166505)

[Table S9: Count of One Health Components in Scoping Review Studies	18](#_Toc239166506)

[Table S10: Counts of Infectious Disease Topics Researched in Scoping Review Studies	19](#_Toc239166507)

[Graph Network Statistics	20](#_Toc239166508)

[Supplement References	22](#_Toc239166509)

# Supplemental Methods

## Table S1: Scoping Review Topical Screening Criteria

| **Category** | **Explanation** | **Rationale for Exclusion** |
| --- | --- | --- |
| Cross-Sectional, Prevalence, and Intervention Evaluation | These studies focus on establishing the association between One Health pillars, a pathogen, and/or risk factors. Many One Health studies focus on establishing baseline disease prevalences. | Though statistical tests are often conducted for these studies, the research activity focuses more on data collection than constructing a mathematical framework. |
| Reviews | Studies that use bibliographic or literature review techniques. | Despite excluding reviews in the search criteria, some review studies are not marked as such in PubMed. |
| Perspectives | Editorial articles or policy recommendations. | These papers have relevance to the topic but do not propose a specific mathematical framework. |
| Laboratory-based | Studies that focus on animal-models, vaccine development, or other pure laboratory research. | These papers do not use mathematical modelling but rather focus on laboratory methods. |
| One Health Coordination, Conceptual Analyses, and Stakeholder Research | As an approach for public health coordination, many studies focus on analyzing the One Health “model” in public health practice. | These studies evaluate One Health practice through qualitative or other non-mathematical approaches. |
| Irrelevant or unrelated | Studies that use a One Health definition that does not align with our study’s. One Health is sometimes included in a study’s implications rather than in its methodology. | These studies are outside the scope of One Health. |
| Modelling studies with no food systems relevance | One Health modelling studies with no direct involvement with livestock, food, or agricultural environments. | Outside the scope of the purpose of the review which are One Health modelling studies with food systems relevance. |

## Table S2: Data Extraction Criteria for Modelling Domains

| **Modelling Domains** | **Explanation** |
| --- | --- |
| Epidemiologic | Models that evaluate the distribution and/or determinants of health or disease. |
| Bioinformatics | Models that analyze the relatedness of genomic information. |
| Simulation | Models that simulate data, processes, or scenarios. Can be deterministic or stochastic. |
| Network | Models that analyze data within a graph structure (nodes and edges). |
| Ecological | Models that evaluate the distribution of living organisms in their respective niches. |
| Economic | Models that evaluate the distribution of resources or pertain to the production of goods and/or services. |
| Spatial | Models that evaluate spatial effects as an independent variable or distribution as a dependent variable. |
| Time Series | Models that evaluate time as an independent variable. |
| Risk Assessment | Models that focus on the systematic risks of exposure or infection. |
| Environmental | Models that analyze environmental processes, such as water, air, soil, climate, and/or land changes. |

## Table S3: Data Extraction Criteria for Data Types

| **Data Types** | **Explanation** |
| --- | --- |
| Genomic | DNA, RNA, or other nucleic acid sequences. |
| Geospatial | Any data related to physical location attributable to geospatial coordinates, sub-national administrative regions, or rasterized grids. |
| Immunological | Data related to immunological response to an antigen (presumably the infectious agent). |
| Ecological | Data related to the relationship between an organism and its ecological niche or distribution. |
| Symptomatic | Data related to disease symptoms. |
| Disease Counts | Data on diagnosed or attributed illnesses/infections. |
| Risk Factors | Data on exposures or determinants of disease status. |
| Environmental | Data on environmental processes, such as water, air, soil, climate, and/or land changes. |
| Agricultural | Data related to agricultural production, food distribution, or farming infrastructure. |
| Economic | Data related to the distribution of resources, including prices, supply, demand, investment, and labor. |

## Table S4: Data Extraction Criteria for One Health Pillars

| **One Health Pillar** | **Explanation** |
| --- | --- |
| Human | Homo sapiens |
| Wildlife | Animals living in ecological niches outside of agricultural production or domestic households. |
| Livestock | Animals raised for the production of food, goods, or services. |
| Domestic Animals | Animals kept in households for companionship and not intended for food production purposes. |
| Environment | Pathogen persistence or proliferation in water, air, soil, and other environmental areas. |
| Food | Pathogen persistence or proliferation in food meant for human or animal consumption |
| Aquaculture | Seafood species raised in aquaculture. |

# Supplemental Results

## OH-JPA Activities Relevant to the Food System

Actions and activities found to be most relevant for agri-food systems modeling in the OH-JPA

- Action Track 1: Enhancing One Health Capacities to Strengthen Health Systems
  - AT1.1 Establish Foundations for One Health Capacities
    - 1.1.4 Define processes and develop methodologies for assessing countries’ vulnerabilities to One Health challenges, and link with appropriate evidence-based preparedness and response capabilities to tackle risks from emerging and re-emerging pathogens and diseases, leading to improvements in the health of humans, animals, plants and the environment at a systems level
- Action Track 2: Reducing The Risks from Emerging and Re-Emerging Zoonotic Epidemics and Pandemics
  - AT2.1 Understanding Drivers of Emergence, Spillover and Spread of Zoonotic Pathogens
    - 2.1.4 Indicator framework for baselines and databases, triggers
  - AT2.2 Identify and Prioritize Evidence-Based Upstream Interventions for The Prevention Of Zoonoses
    - 2.2.2 land-use planning and biodiversity, risk assessments
    - 2.2.3 habitat degradation, biodiversity protection in food systems, indicators
    - 2.2.7 evidence-based prevention/control of zoonoses along value chains, livestock,
  - 2.3 Strengthen One Health Surveillance, Early Warning, And Response
    - 2.3.1 Develop operational tools and resources to conduct targeted One Health surveillance at human-animal ecosystem interfaces and a mechanism for multi-sectoral data sharing, per Quadripartite/OHHLEP guidance, supported by robust regulatory frameworks
    - 2.3.2 Develop guidance on progressive control and management pathways that apply a One Health approach in strengthening biosecurity for existing and potentially re-emerging zoonotic diseases (such as zoonotic influenza viruses, MERS-CoV, SARS-CoV-2, Ebola and Rift Valley fever) and support countries with implementation
    - 2.3.4 Develop a pathogen monitoring framework for wildlife and the environment, including in wildlife habitats, on farming and trade routes and along the wild meat and products value chain, and support countries with implementation
- Action Track 3: Controlling And Eliminating Endemic Zoonotic, Neglected Tropical and Vector-Borne Diseases
  - AT3.1 Enable Countries to Implement Neglected Zoonotic Disease
    - 3.1.3 Agricultural water and waste management
- Action Track 4: Strengthening The Assessment, Management and Communication of Food Safety Risks
  - 4.1 Strengthen One Health Approach in National Food Controls Systems and Food Safety Coordination
    - 4.1.1 Food safety throughout value chain, connections in food safety activity
  - AT4.2 Improve Food System Data and Analysis, Scientific Evidence and Risk Assessment
    - 4.2.1 One Health into food safety risk assessments
    - 4.2.2 Food safety risk assessments through food systems lens
  - AT4.3 Foster the Adoption in Foodborne Disease Surveillance Systems and Research
    - 4.3.1 Antimicrobial resistant foodborne pathogens
    - 4.3.2 Food safety data integrations and open data
    - 4.3.3 Surveillance at intersection of human-animal-plant-environment interface for attributing food sources under One Health frame
    - 4.3.4 Research of transmission of foodborne pathogens at interface
- Action Track 5: Curbing the silent pandemic of AMR
  - AT5.1 Strengthen Country Capacity and Capability to Control AMR
    - 5.1.2 Integrated surveillance
- Action Track 6: Integrating the environment into One Health
  - AT6.1 Protect Restore, And Prevent the Degradation of Ecosystems and The Wider Environment
    - 6.1.1 knowledge products on anthropogenic factors leading to environmental degradation
    - 6.1.3 Transition to sustainable agriculture and livestock production
  - AT6.2 Mainstream the Health of The Environment and Ecosystems into The One Health Approach
  - AT6.3 Integrate Environmental Knowledge, Data and Evidence in Decision-Making
    - 6.3.1 Map interoperability between databases
    - 6.3.2 Linkages for environmental data in risk modeling
    - 6.3.3 Joint information management systems

## Table S5: Food System - One Health Priorities with OH-JPA Cross-Reference

| **Food System - One Health Priorities** | **Exposures and Population** | **Example Pathogens/Disease** | **Common Research Approaches** | **OH—JPA Cross-Referenced Action Tracks** |
| --- | --- | --- | --- | --- |
| **Food Safety** | Follow fecal-oral infection route. Global issue, but pronounced morbidity and mortality in developing countries. | Salmonella spp., E. coli, Cyclospora, Norovirus, Listeria monocytogenes. Physical and chemical hazards are also major concerns. | Risk assessment, case-control, randomized controlled trials (RCTs), genomic epidemiology. | 2.2, 2.3, 3.1, 4.1, 4.2, 4.3, 6.3 |
| **Zoonotic Disease / Emerging Infectious Disease / Vector-borne** | Exposure may be fecal-oral, respiratory, fomites, or vector-based. EID also concerned with pandemic preparedness as well as selected agents. Categories sometimes treated distinctly, despite overlap. | Zoonoses include Avian Influenza, Coronaviruses, Brucellosis, Ebola, Rabies.  EIDs include Monkeypox, Nipah. Select agents include Anthrax, Smallpox. Vector-borne include Malaria, Leishmania, African trypanosomiasis, Babesiosis. | Compartment models, case-control, cohort, RCTs, risk assessment, epi-ecologic models, genomic epidemiology, Bayesian methods, simulations, geospatial. | 1.1, 2.1, 2.2, 2.3, 3.1, 6.1, 6.2, 6.3 |
| **Neglected Tropical Disease** | Illnesses often have a significant economic impact and simple, inexpensive interventions. Various exposure types. Many are parasitic. | Lymphatic filariasis, Onchocerciasis, Schistosomiasis, Trachoma, Guinea worm, Chagas disease. | Same as zoonotic. Economic modeling regarding intervention effectiveness. | 1.1, 3.1, 6.3 |
| **Water Hygiene** | Fecal-oral route. Similar to NTDs, infrastructure and economic dimensions to intervention. | Water hygiene may be concerned with chemical/toxin and/or waterborne pathogens. | Hydrological modeling, geospatial, machine learning, image processing, toxicological assessments. | 3.1, 4.2 |
| **Surveillance** | Surveillance are public health information systems tasked with signal detection of emergent pathogens. | Can be any, but largely focus on infectious disease with outbreak, pandemic, or bioterror potential. | IT infrastructure, stream processing, interoperability, and decision analytics | 1.1, 2.3, 4.3, 5.1, 6.3 |
| **Pandemic Preparedness and Response** | Multiple exposure types, but emphasis on widespread immune vulnerability and significant morbidity and mortality. | Significant cross-over with zoonoses/EID. Monitoring of pathogen evolution for pandemic potential and as of yet unknown agents. | Gain of function studies, compartmental models, simulations, Bayesian hierarchical, machine learning, genomic epidemiology, economic. | 1.1, 2.1, 2.3, 6.3 |
| **Antimicrobial Resistance** | Primarily bloodstream infections and respiratory diseases. Fecal-oral route also possible. | E. coli, Acinetobacter, Pseudomonas, K. Pneumoniae, Staphylococcus aureus. | Genomic epidemiology, compartmental models, simulations | 4.3, 5.1, 6.3 |

## Table S6: Group of 20 National One Health Initiatives

| **Country/Entity** | **Lead Ministry/Agency** | **Governance** | **Responsibilities and Disease Initiatives** | **Resources with Links** |
| --- | --- | --- | --- | --- |
| Argentina | Administración Nacional de laboratorios e Institutos de Salud (National administration of laboratories and institutes of health.) | Unit is called El Instituto Nacional de Medicina Tropical, founded in 2011 | Research, Capacity building, Advisement, Information dissemination, and Surveillance  National Strategy on Antimicrobial Resistance from SENASA,  Eco-epidemiology, especially for tropical and sub-tropical diseases. | [One Health Office](https://www.argentina.gob.ar/salud/inmet/mision)  [Press release](https://www.argentina.gob.ar/senasa/resistencia-antimicrobiana) |
| Australia | Australian Centre for Disease Control,  with partners Department of Agriculture, Fisheries and Forestry  Department of Climate Change, Energy, the Environment and Water  Department of Foreign Affairs and Trade. | Integrated into core functions, One Health, Climate, and Environment Branch, One Health Unit. Mention OH-JPA. | Collaboration – by improving collaboration between different sectors, to address complex health issues such as zoonoses control and antimicrobial resistance  Capacity building – by building on lessons from the experiences of recent outbreak responses in Australia, including Japanese encephalitis virus and mpox  Coordination – by working with other organisations to better identify emerging threats and deliver faster and more effective responses  Communication – by engaging with our partners to identify, develop and deliver relevant One Health messages for our stakeholders and the Australian public.  (from AuCDC website)  Identify potential and emerging zoonoses sooner  Reduce their impacts to human–animal–environmental health and national economies  Improve food safety and security  Protect biodiversity and the world’s ecosystems.  (from AuCDC website) | [One Health Landing Page](https://www.cdc.gov.au/topics/one-health)  [CDC Organizational Chart](https://www.cdc.gov.au/about-us/our-leadership-and-governance) |
| Brazil | Ministry of Health (Ministério da Saúde), Secretariat of Health Surveillance and Environment | Since 2019, Technical Group of One Health (Grupo Técnico de Uma Só Saúde). Legislation in 2024. Mention OH-JPA | Translated from Actions of Ministry of Health:   - I - prepare and review the National Action Plan of One Health; - II - support, monitor and propose adjustments to the implementation of the National Plan of Action of One Health; - III - articulate with States and Municipalities with a view to guiding interfederative and multi-sectoral measures for the implementation of the National Plan of Action of One Health; - IV - technically advise the Brazilian Government on domestic and international agendas on the subject; and - V - support the development of studies and research on the subject.   Translated from One Health Landing Page:   - Surveillance, prevention and control of zoonoses and neglected tropical diseases and vector-borne diseases; - Qualification of prevention, preparedness and response to epidemics and pandemics; - Promoting food safety and processing of agri-food systems; - Combating antimicrobial resistance; - Awareness of the relationships between human, animal, plant and environmental health; - Protection of biodiversity and improvement of ecosystem management; - Confronting and adapting to climate change; - Control of chemical, biological and physical contaminants, among other topics. | [Actions of the Ministry of Health](https://www.gov.br/saude/pt-br/assuntos/saude-de-a-a-z/u/uma-so-saude/acoes-do-ministerio-da-saude)  [One Health Landing Page](https://www.gov.br/saude/pt-br/assuntos/saude-de-a-a-z/u/uma-so-saude)  [Ministry of Agriculture and Livestock Landing Page](https://www.gov.br/agricultura/pt-br/assuntos/defesa-agropecuaria/uma-so-saude/programas) |
| Canada | Federal Health Portfolio | Distributed throughout many centers. No central coordinative unit. Mention OH-JPA. | From One Health Landing Page:   - conducting monitoring, surveillance, laboratory diagnostics, modelling, risk assessments - using data collection and analysis to help identify potential outbreaks and allow for timely interventions, as well as active prevention and communications activities - working with partners within Canada and abroad to support pan-Canadian and international surveillance initiatives - ensuring robust laboratory diagnostics to provide timely detection of infectious diseases - using modelling to assess the potential spread and impact on infectious diseases and other health threats to help inform preparedness and response - undertaking risk assessments to evaluate potential threats to human, animal, plant and environmental health - engaging with partners across the human-animal-plant-environment interface - collaborating and coordinating activities and actions with national and international partners including provincial and territorial government agencies, academia, non-government organizations and industry partners - engaging with international standard setting bodies to advocate for inclusion of human, animal, plant and environmental health considerations - coordinating communications on preventing, preparing for and responding to threats - providing clear and timely communications to help people in Canada make well-informed decisions to take preventive measures against threats - building on preparedness activities to respond effectively to threats, including those with pandemic potential - developing health emergency and pandemic prevention, preparedness and response plans, scenarios and relevant infectious disease guidance with One Health partners - continuing research and innovation to improve understanding of threats, including infectious diseases, and developing new tools and strategies for preparedness, prevention and control - monitoring new and evolving science to help inform current and future One Health activities - participating in science coordination and engagement with experts to enable an evidence-based approach to threats - antimicrobial resistance, when germs like bacteria, viruses, fungi and parasites no longer respond to medicines used to prevent or treat the infections they cause - zoonotic diseases, which are diseases that can spread between animals and people - wildlife health, which includes monitoring diseases and their impacts on populations of wild animals - food safety and security, ensuring safe and stable food systems, from farm to fork, for people and animals - plant and environmental health threats such as:   - contaminated water   - impacts of extreme weather events   - habitat changes, such as deforestation   - introduction and spread of plant pathogens, pests and weeds | [One Health Landing Page](https://www.canada.ca/en/health-canada/services/science-research/one-health.html) |
| China | Little public information |  | Complex governance within integrated health governance system.[1] |  |
| France | Ministry of Health (Ministre de la Santé, des Familles, de l’Autonomie et des Personnes handicapées) | Interministrial Program,  Mention OH-JPA | Translated from AMR Action Plan:   - Engage Stakeholders - Develop research - Reinforce coordination of integrated surveillance tools - Protect existing antibiotic effectiveness and improve their utilization. - Develop international cooperation - Antimicrobial Resistance | [Landing Page](https://sante.gouv.fr/grands-dossiers/article/one-health-l-approche-globale-une-seule-sante)  [AMR Action Plan](https://www.ecologie.gouv.fr/sites/default/files/documents/feuille_de_route_interministerielle_2024_-_2034_antibioresistance_et_resistance_aux_antimicrobiens_-sept_2024.pdf) |
| Germany | Distributed approach throughout several agencies and government institutes: Robert-Koch-Institut, Helmoltz Institut für One Health, Friedrich Loeffler Institut, One Health Platform | Mentions OH-JPA | - Research coordination - Collaboration - Capacity building - Antimicrobial Resistance - Zoonoses | [One Health Platform](https://onehealthplatform.net/en/home) |
| India | Department of Health Research | National One Health Mission (founded in 2024), cross-ministerial initiative | From National One Health mission:   - Capacity building, collaboration, communication and storage of potential high- risk pathogens - Creation of a national network of existing and upcoming high-risk pathogens laboratories (BSL-3/4) labs across departments and keeping them functional through continuous R&D and use during outbreak response - Developing and assessing the feasibility of various AI tools, NGS, diagnostic algorithms for detection/discovery of emerging novel pathogens in humans, animals and environment and establish metagenomic pipelines for novel pathogen discovery. - Develop protocols, tools and framework for integrated disease surveillance at the animal-human-environment interface for emerging diseases. - Plug and play vaccine platforms, diagnostics, capacity for development of high quality therapeutic monoclonal. - Develop analytic capability to derive better insights from the data and information of human, livestock and wildlife sectors - To build an integrated disease control and pandemic preparedness system in India by bringing human, animal and environmental sectors together for better health outcomes, improved productivity and conservation of biodiversity. | [National One Health Mission](https://www.icmr.gov.in/national-one-health-mission) |
| Indonesia | ASEAN One Health Joint Plan of Action announced, but no other information found | | | |
| Italy | Ministero della Salute (Ministry of Health), Istituto Superiore di Sanità | Department of Human Health, Animal Health, and Ecosystem (One Health) and International Relations, ISS One Health (OH) group | Translated from One Health Landing Page:   - identification, definition, evaluation, information and promotion of correct lifestyles, in relation to the ecosystem, the living environment and the work environment - exercise of state competences in nutrition, food and food education - food safety risk assessment - animal health, veterinary medicines and animal welfare - coordination and financing of experimental zooprophylactic institutes and supervision of the same - research and experimentation in the food and veterinary sector - protection of the environment and ecosystem, living conditions and well-being of people - Cross-border assistance - International coordination | [One Health Landing Page](https://www.salute.gov.it/new/it/ministero/dipartimento-salute-umana-salute-animale-ecosistema-one-health-rapporti-internazionali/)  [Institute for Superior Health](https://www.iss.it/en/one-health) |
| Japan | Ministry of Health, Labour, and Welfare, with Ministry of Agriculture, Forestry and Fisheries, and Ministry of the Environment | Interministerial, mentions OHJPA | Translated from One Health Landing Page:  **Ministry of Health, Labour and Welfare (Human focus)**  **Monitoring:** Physicians and veterinarians report human disease occurrences and animal pathogen carriage under the Infectious Disease Act; research conducted through MHLW and AMED studies (including zoonoses)  **Source control:** Eradication/disinfection of infection sources (ticks, mosquitoes, rats, etc.); mandatory rabies vaccination and municipal registration of dogs (Rabies Prevention Act); slaughterhouse inspections and food hygiene measures  **Public awareness:** Outreach via websites, posters, and symposiums; dissemination of professional guidelines to sellers and related parties  **Import/Quarantine:** Import bans on pet monkeys, bats, raccoon dogs, masked palm civets, etc.; verification of sanitary certificates for imported animals; on-site inspections of monkey breeding facilities in exporting countries  **Ministry of Agriculture, Forestry and Fisheries (Livestock focus)**   - Monitoring of livestock/poultry disease outbreaks (Livestock Infectious Disease Prevention Act) - Research on livestock diseases (including zoonoses) - Promotion of hygienic management for livestock/poultry farming - Information provision to livestock farmers and related parties - Import/export quarantine for dogs (Rabies Prevention Act) - Import quarantine for research/exhibition monkeys (Infectious Disease Act) - Import/export quarantine for livestock, poultry, and livestock products   **Ministry of the Environment (Wildlife & environment focus)**   - Avian influenza (wild bird) surveys - Hygiene management of animal dealers - Microchip (MC) registration for dogs and cats (Animal Welfare Act) - Climate change countermeasures - Wildlife protection and management - Public outreach to animal owners and the general public   Antimicrobial Resistance, Zoonoses | [One Health Landing Page](https://www.mhlw.go.jp/stf/seisakunitsuite/bunya/0000172990.html) |
| Mexico | All governmental health institutions (CENAPRECE, DGE, DGPS y COFEPRIS), de Salud Animal (SENASICA – Dirección en Jefe, DGSA, CENASA y CENAPA), de Ambiente (DGVS y CONANP), | Interministerial, mentions OHJPA | Zoonoses (Rabies) mentioned but few other details | [One Health Landing Page](https://una.salud.gob.mx/) |
| Russia | No public resources found | | | |
| Saudi Arabia | No public resources found | | | |
| South Africa | No public resources found | | | |
| South Korea | National Institute of Health | One Health AMR , also mentions OHJPA, council at Korea CDC | - Research - Coordination - Laboratory capacity - Antimicrobial resistance | [AMR Info Page](https://nih.go.kr/nohas/en/common/reschField/ctg01/rf-intro.do) |
| Turkey | No public resources found | | | |
| United Kingdom | No coordinative body found | | | |
| United States of America | Headed by Centers for Disease Control and Prevention,  with coordinative structures in Department of Agriculture, Environmental Protection Agency, and Department of Interior | Founded in 2009 as a permanent office by statute. Mention OH-JPA. | From CDC website:   - Lead One Health strategy - Coordinate partners to operationalize and strengthen One Health in the United States - Advance One Health globally through development of tools and trainings for capacity building for global health security - Leverage CDC's expertise and engage partners to tackle One Health issues - Provide technical expertise at the human-animal-environment interface, including during public health emergencies - Fill information gaps on the control and prevention of pet-associated zoonoses through the Healthy Pets, Healthy People program - Zoonotic and EID - Pandemic preparedness and response - One Health emergencies at human-animal-environment interface - One Health pan-respiratory disease surveillance - Global Health security and capacity building - Strengthen One Health coordination in the US - Strategic One Health partnerships - Prevent zoonoses shared between people and pets | [Centers for Disease Control and Prevention](https://www.cdc.gov/one-health/about/index.html)  [United States Department of Agriculture](https://www.aphis.usda.gov/one-health)  [Environmental Protection Agency](https://www.epa.gov/one-health)  [Department of Interior](https://www.doi.gov/emergency/one-health) |
| European Union | the European Environment Agency (EEA)  the European Centre for Disease Prevention and Control (ECDC)  the European Chemicals Agency (ECHA)  the European Food Safety Authority (EFSA)  the European Medicines Agency (EMA) | Cross-agency One Health Task Force | From Landing Page:   - Facilitate strategic coordination in the - implementation of the One Health - approach; - Promote research coordination and One - Health-driven agenda setting; - Enhance capacity building on One Health; - Strengthen One Health communication - and stakeholders' engagement; - Support the development of partnerships - through joint One Health activities | [Landing page](https://health.ec.europa.eu/one-health/cross-agency-one-health-task-force_en) |

## Table S7: Counts of Data Types Used in Scoping Review Studies

| **Data Type(s)** | **Studies** |
| --- | --- |
| Environmental | 62 |
| Agricultural | 54 |
| Disease Counts | 50 |
| Risk Factors | 44 |
| Geospatial | 36 |
| Genomic | 32 |
| Ecological | 31 |
| Economic | 22 |
| Immunological | 10 |
| Symptomatic | 6 |

## Table S8: Count of Modelling Domains Used in Scoping Review Studies

| **Modelling Domain** | **Studies** |
| --- | --- |
| Epidemiologic | 82 |
| Environmental | 50 |
| Spatial | 38 |
| Bioinformatics | 32 |
| Ecological | 32 |
| Risk Assessment | 31 |
| Time Series | 20 |
| Economic | 18 |
| Simulation | 9 |
| Network | 5 |

## Table S9: Count of One Health Components in Scoping Review Studies

| **One Health Component** | **Studies** |
| --- | --- |
| Livestock | 87 |
| Human | 79 |
| Wildlife | 32 |
| Environment | 30 |
| Food | 21 |
| Domestic Animals | 13 |
| Aquaculture | 4 |

## Table S10: Counts of Infectious Disease Topics Researched in Scoping Review Studies

| **Disease** | **Studies** |
| --- | --- |
| Antimicrobial Resistance | 45 |
| Generic | 16 |
| Influenza A virus (avian strains, e.g. H5N1) | 11 |
| Escherichia coli (STEC, e.g. O157:H7) | 11 |
| Salmonella spp. | 9 |
| Leptospira spp. | 7 |
| Brucella spp. | 7 |
| Schistosoma spp. | 5 |
| Bacillus anthracis | 4 |
| Campylobacter jejuni | 4 |
| Crimean-Congo hemorrhagic fever virus | 4 |
| Trypanosoma cruzi | 3 |
| Fasciola hepatica | 3 |
| Coxiella burnetii | 3 |
| Japanese encephalitis virus | 2 |
| Acinetobacter baumannii | 2 |
| Mycobacterium bovis | 2 |
| Rabies virus (Lyssavirus) | 2 |
| Taenia solium | 2 |
| Rift Valley fever virus | 2 |
| Leishmania spp. | 2 |
| Echinococcus granulosus / E. multilocularis | 2 |
| Hantavirus (Sin Nombre, Seoul, Andes, etc.) | 1 |
| Candida spp. | 1 |
| Vibrio spp. | 1 |
| Anaplasma phagocytophilum | 1 |
| Trypanosoma brucei | 1 |
| Theileria orientalis | 1 |
| Aspergillus Spp. | 1 |
| Streptococcus pneumoniae | 1 |
| Staphylococcus spp. | 1 |
| Babesia microti | 1 |
| Bluetongue virus | 1 |
| SARS-CoV-2 | 1 |
| Rickettsia rickettsii | 1 |
| Enterocytozoon bieneusi | 1 |
| Clonorchis sinensis | 1 |
| Plasmodium falciparum | 1 |
| Pesticides | 1 |
| Paragonimus westermani | 1 |
| Orientia tsutsugamushi | 1 |
| Opisthorchis viverrini | 1 |
| Mycobacterium avium | 1 |
| Mental health | 1 |
| Ehrlichia ruminantium | 1 |
| Aeromonas hydrophila | 1 |
| West Nile virus | 1 |

## Graph Network Statistics

***Graph Network Statistics for Data Types***

Centrality

{'Symptomatic': 0.4444444444444444,

'Disease Counts': 1.0,

'Risk Factors': 1.0,

'Genomic': 0.6666666666666666,

'Environmental': 0.8888888888888888,

'Agricultural': 1.0,

'Economic': 0.6666666666666666,

'Immunological': 0.7777777777777777,

'Ecological': 0.8888888888888888,

'Geospatial': 0.8888888888888888}

Approximate Current Flow Betweenness Centrality

{'Symptomatic': 0.06714092140921403,

'Disease Counts': 0.1746311352002409,

'Risk Factors': 0.1610809996988859,

'Genomic': 0.08270099367660352,

'Environmental': 0.14783197831978326,

'Agricultural': 0.16619617585064747,

'Economic': 0.07551189400782901,

'Immunological': 0.10444143330322189,

'Ecological': 0.14252484191508594,

'Geospatial': 0.12086344474555863}

***Graph Network Statistics for Modelling Domains***

Centrality

{'Bioinformatics': 0.8888888888888888,

'Epidemiologic': 1.0,

'Time Series': 0.7777777777777777,

'Risk Assessment': 1.0,

'Ecological': 0.8888888888888888,

'Economic': 1.0,

'Environmental': 0.8888888888888888,

'Simulation': 0.6666666666666666,

'Network': 0.5555555555555556,

'Spatial': 1.0}

Approximate Current Flow Betweenness Centrality

{'Bioinformatics': 0.11629904204039293,

'Epidemiologic': 0.14846886631277081,

'Time Series': 0.1207780370980536,

'Risk Assessment': 0.13546433583501127,

'Ecological': 0.10343446824089327,

'Economic': 0.16019319360546716,

'Environmental': 0.11287677710659588,

'Simulation': 0.0926040331929953,

'Network': 0.08771279516749036,

'Spatial': 0.102093248520349}

***Graph Network Statistics for One Health Component***

Centrality

{'Human': 0.8333333333333333,

'Food': 1.0,

'Wildlife': 0.8333333333333333,

'Livestock': 0.8333333333333333,

'Domestic Animals': 0.8333333333333333,

'Environment': 0.8333333333333333,

'Aquaculture': 0.16666666666666666}

Approximate Current Flow Betweenness Centrality

{'Human': 0.11666666666666668,

'Food': 0.40833333333333305,

'Wildlife': 0.08750000000000001,

'Livestock': 0.18958333333333338,

'Domestic Animals': 0.13125000000000003,

'Environment': 0.14583333333333337,

'Aquaculture': 0.0}

# Supplement References

1. Li X, Zhang Y, Zhang Q, Liu J, Zhu Z, Feng X, et al. Strategy and mechanism of One Health governance: case study of China. Science in One Health. 2025;4:100098. https://doi.org/10.1016/j.soh.2024.100098.
